# Plant Behaviour, Adaptive Plasticity and Evolution: Reassessing the Debate on Plant Intelligence

**DOI:** 10.3390/biology15181632

**Published:** 2026-09-16

**Authors:** Claudia Rosciglione

**Affiliations:** Dipartimento di scienze umanistiche (SUM), University of Palermo, 90128 Palermo, Italy; claudia.rosciglione@unipa.it

**Keywords:** plant behaviour, plant cognition, evolution, phenotypic plasticity, plant communication, adaptation, plant intelligence, philosophy of biology

## Abstract

In recent decades, plant sciences have profoundly transformed our philosophical understanding of plants, moving beyond the traditional view of the plant organism as a passive, mechanical entity devoid of agency. Contemporary research into plant physiology, chemical communication and adaptive strategies has helped to outline a theoretical framework: plants emerge as complex systems, capable of environmental perception, signal integration, physiological memory and flexible responses to stimuli. Recent findings have stimulated an extensive interdisciplinary debate on whether plants should be attributed forms of cognition or even intelligence. This paper critically analyses the relationship between plant behaviour and evolutionism, examining the concepts of adaptation, communication and plant intelligence in the light of evolutionary biology, the philosophy of mind and the epistemology of the life sciences. This review examines how the case of plants helps to redefine traditional categories such as organisms, behaviours, subjectivities and cognition. It opens up new theoretical perspectives for a non-anthropocentric philosophy of life.

## Simple Summary

Plants have traditionally been regarded as passive organisms that simply respond to environmental conditions. However, recent research has shown that they can perceive a wide range of external signals, communicate through chemical and electrical pathways, modify their development according to changing environments, and retain physiological information from previous experiences. These discoveries have challenged traditional views of plant life and stimulated a growing interdisciplinary debate on concepts such as plant behaviour, adaptation, memory and intelligence. This review examines current evidence from plant biology together with perspectives from evolutionary theory and the philosophy of biology. Rather than asking whether plants are intelligent in the same sense as animals, the review explores how plant behaviour can be understood as the outcome of highly integrated and adaptive biological processes that do not require a nervous system. The review also discusses the conceptual and methodological challenges involved in applying cognitive terminology to plants and argues that plant research offers valuable insights into the evolution of complex adaptive systems. By integrating biological evidence with philosophical analysis, this review contributes to a broader and less anthropocentric understanding of living organisms.

## 1. Introduction

For much of the history of Western biology, plants have been regarded as essentially passive organisms, incapable of behaviour in the sense attributed to animals. This conception stems from a long philosophical tradition which, beginning with Aristotle, has rigidly distinguished vegetative life from sensory and rational life. Within this hierarchy, the plant world was associated solely with the functions of nutrition, growth and reproduction, whilst behaviour, intentionality and perception were regarded as the prerogatives of animals. This paradigm was further reinforced by modern mechanistic thought. Descartes equated non-human organisms with biological automata, whilst nineteenth-century physiology interpreted plant behaviour in exclusively chemical–mechanical terms. Within this epistemological framework, the very idea of plant behaviour appeared problematic: plants grew but did not act; they reacted but did not decide. This approach has also profoundly influenced modern natural sciences. For a long time, behaviour was defined in relation to voluntary movement, the presence of a nervous system and the capacity for centralised cognitive processing. Plants, lacking a brain and incapable of locomotion, thus appeared to be excluded from the domain of ethology.

However, as early as the 19th century, some observations began to challenge this traditional view. Charles Darwin, in his book *The Power of Movement in Plants*, published in 1880, observed that the root tips appeared to perform a coordinating function analogous, at least in a functional sense, to that of a nerve centre in lower animals. Although this insight did not gain immediate acceptance, it anticipated a theme destined to re-emerge forcefully in contemporary debate. In recent decades, advances in plant physiology, molecular biology, behavioural ecology and comparative neuroscience have profoundly altered the theoretical framework. Plants are now recognised as organisms capable of perceiving complex environmental signals, integrating information from different sources and modulating their responses according to the context. Such capabilities do not necessarily imply consciousness or intentionality in the human sense, but they do highlight the inadequacy of a purely mechanistic conception of the plant world. Rather, the plant emerges as a dynamic system, capable of developing sophisticated adaptive strategies through regulatory processes spread throughout the entire organism. These phenomena have given rise to a lively interdisciplinary debate. Some authors speak of “plant neurobiology”, whilst others prefer more cautious terms such as “adaptive plasticity” or “distributed behaviour”. The empirical investigation of plant cognition primarily falls within the remit of cognitive science. However, extending concepts such as cognition, intelligence and behaviour to organisms lacking nervous systems raises important conceptual questions in the philosophy of biology. This is particularly the case when it comes to the scope and meaning of these categories. To what extent is it legitimate to use cognitive categories to describe the plant world? Does intelligence necessarily require a brain? Do non-neural forms of cognition exist? The issue is not merely one of terminology, but touches on deeper questions concerning the very definition of behaviour, learning and adaptation. In this sense, the study of plants now constitutes one of the most fascinating fields for interdisciplinary reflection on the foundations of contemporary biology. This review addresses these issues from an evolutionary and philosophical perspective. After examining the concept of plant behaviour, it will analyse the adaptive dynamics of plants, the mechanisms of intra- and interspecific communication, and the debate on plant intelligence. Finally, it will discuss the epistemological and ontological implications of this research for contemporary philosophy. I argue that contemporary plant biology does not support the attribution of intelligence to plants in the strong mentalistic sense, involving conscious, representational or deliberative problem-solving capacities, as are commonly attributed to animals with nervous systems. Instead, it shows that plant behaviour is best understood as a form of distributed adaptive regulation shaped by evolution. I argue that the main philosophical contribution of plant research is not to show that plants possess minds comparable to those of animals, but rather to necessitate a conceptual revision of categories such as behaviour, cognition, and agency. Throughout this review, I adopt the following operational strategy: first, I describe the observable biological phenomenon; then, I identify its underlying mechanism; only afterwards do I discuss whether cognitive terminology is conceptually justified. This review defends a non-representational and broadly enactive position, which is constrained by evolutionary and operational criteria. It is not assumed that every instance of adaptive self-maintenance is cognitive. Cognition is only attributed where there is context-sensitive integration of multiple signals, flexible selection among alternative responses, retention or use of information beyond the immediate stimulus, and organism-level adjustment that cannot be adequately characterised by a simple stimulus–response description. These criteria are intended as operational thresholds rather than as evidence of consciousness or mental representation. They therefore only support a minimal, non-conscious conception of plant cognition.

## 2. Plant Behaviour: Phenotypic Plasticity and Chemical Communication

Before addressing the topic of plant behaviour, it is necessary to clarify what is generally meant by “behaviour” in a biological context. Traditionally, the concept has been developed primarily in relation to animals and their interactions with the environment. From the classical perspective of ethology, behaviour was generally defined as the set of observable actions through which an organism responds to external stimuli [1,2,3]. However, such a definition tends to favour organisms possessing mobility and a nervous system, thereby implicitly excluding the plant kingdom. For this reason, numerous contemporary scholars have proposed a redefinition of the concept in broader and more functional terms.

As I have already mentioned in the introduction, Darwin’s theory of evolution provides the fundamental conceptual framework for understanding plant behaviour [4]. In *On the Origin of Species*, Darwin already attached great importance to plant movements and the sensitivity of roots, which he regarded as structures capable of orienting the organism in space. In the book *The Power of Movement in Plants* (1880), written with Francis Darwin, the English naturalist observed that the root apex behaves “like the brain of a lower animal”, coordinating the responses of the entire plant [5] (p. 543). Darwin’s insight anticipated a perspective that is central today: behaviour does not necessarily coincide with locomotion. In evolutionary biology, behaviour can be defined as an organised sequence of plastic responses to environmental stimuli, geared towards survival and reproduction. From this perspective, plants too possess behavioural repertoires. Plant adaptation manifests itself through extremely sophisticated morphological, physiological and chemical strategies. Plants regulate root growth according to nutrient availability; they orient their leaves and stems in response to light gradients; they activate chemical defences against herbivores and pathogens; and they adjust their reproductive timing in response to climatic conditions. From an evolutionary perspective, these capabilities do not imply conscious intent. Rather, they represent the result of selective processes that have favoured biological systems capable of responding flexibly to environmental pressures. However, the degree of integration and plasticity of these responses raises theoretical questions about the nature of behaviour itself. In a functionalist framework, behaviour can be understood as any organised response through which an organism modifies its internal state or its relationship with the environment in order to increase its chances of survival and reproduction. Such a definition also allows for the inclusion of plants, whose behaviour is not manifested through locomotion but through processes of growth, physiological regulation and structural modification.

Today it is reasonable to speak of plant behaviour if the term is used in an operational sense: a measurable change in state, growth, movement, chemical release or physiological priority that depends on environmental information and alters the plant’s subsequent interaction with its environment [6]. Plant behaviour is expressed primarily through developmental plasticity. A plant is not a static organism that rigidly follows a predetermined genetic programme; on the contrary, it continually modifies its form in response to environmental conditions. Light, gravity, water availability, soil chemistry, temperature and the presence of competing organisms profoundly influence plant structure and metabolism. Phenomena such as phototropism and gravitropism clearly demonstrate this dynamic. A plant directs its growth towards the light not through immediate movement, but through the differential redistribution of auxins, hormones that regulate cell elongation [7,8]. Similarly, roots modify their development in response to the distribution of water and nutrients in the soil. These responses cannot be regarded as mere mechanical automatisms. They involve the ability to integrate multiple signals and to modulate the response in a context-dependent manner. A single species can develop profoundly different morphological configurations depending on the environment in which it grows.

The concept of phenotypic plasticity takes on decisive importance here. Plants do not follow rigid genetic programmes; they modulate their development in response to ecological conditions. Genetically similar individuals can take on radically different forms depending on the environment. According to Mary Jane West-Eberhard, plasticity represents one of the main adaptive resources of living organisms, as it enables them to produce different phenotypes in response to variable conditions [9]. In the case of plants, this ability is particularly well-developed, precisely because of their immobility. Plants cannot escape from a hostile environment; they must therefore modify themselves. It is precisely this condition that makes plant behaviour so interesting from an evolutionary perspective. At the heart of plant behaviour lies a complex capacity for perception of the environment. Plants are equipped with sophisticated sensory systems that enable them to detect extremely subtle variations in external conditions. They perceive the quality and intensity of light through specialised photoreceptors such as phytochromes and cryptochromes; they detect gravity via intracellular structures called statoliths; they respond to mechanical stimuli such as touch and wind; they recognise chemicals produced by other organisms and even variations in the concentration of nutrients present in the soil. Plants’ perceptual capacity is therefore far more sophisticated than traditionally imagined. These are not merely local reactions, but systems for the continuous monitoring of the environment. Once a stimulus is perceived, the plant must integrate it and transform it into a coherent response. This process takes place through complex biochemical networks involving plant hormones, ionic flows and electrical signals. Auxins, for example, play a fundamental role in regulating growth. In the presence of lateral light, they accumulate on the shaded side of the stem, stimulating differential growth that directs the plant towards the light source [10,11]. Of particular interest is the discovery of electrical signals in plants. Recent studies have shown that plants are capable of generating electrical impulses that propagate through vascular tissues relatively rapidly [2,12]. These signals are not identical to the action potentials found in animal neurons, but they share significant functional similarities. They enable coordination between different parts of the plant, contributing to the regulation of physiological responses. When a leaf is damaged by a herbivore, for example, electrical signals can propagate rapidly throughout the entire organism, triggering defence mechanisms even in regions not directly affected.

All these phenomena suggest that information processing in plants occurs through a decentralised logic. There is no central brain coordinating behaviour; on the contrary, the entire organism participates in regulatory processes. This decentralised organisation constitutes one of the most innovative aspects of contemporary plant biology. It challenges the idea that behavioural complexity necessarily requires the presence of a centralised nervous system. In this sense, plant plasticity has led some philosophers of biology to speak of distributed agency. The plant organism does not possess a single decision-making centre, but coordinates multiple processes through widespread networks of hormonal, electrical and chemical signalling. It appears as a decentralised system of information processing. From this perspective, the notion of plant agency must not be confused with human psychological intentionality. Rather, it refers to an organism’s capacity to maintain its own organisation through selective interactions with the environment. Plants are not conscious beings in the traditional sense, nor are they mere passive automata. This perspective ties in with contemporary theoretical models such as enactivism and complex systems theory, according to which cognition emerges from the dynamic interaction between organism and environment [13]. Life itself can be interpreted as a continuous process of producing biological meaning.

With regard to interactions with the environment, one of the most innovative fields of plant research concerns chemical communication. Plants do not live in isolation but are embedded in extremely complex ecological networks. Their survival depends on their ability to interact with other organisms, including fungi, bacteria, insects and other plants. Numerous studies have demonstrated that plants release volatile organic compounds in response to mechanical damage or herbivore attack. These signals can then modify the defensive responses of neighbouring plants [14,15,16]. These substances can trigger preventive defences in neighbouring individuals. When a plant is attacked by herbivorous insects, for example, it may emit chemical signals that induce other plants of the same species to produce toxic or deterrent metabolites. In some cases, the volatile compounds attract natural predators of the herbivores, forming a complex form of triadic interaction [14]. These phenomena have often given rise to anthropomorphic interpretations: terms such as alarm, cooperation or plant language are used. However, from an epistemological point of view, a distinction must be made between heuristic metaphor and literal description. Plants do not possess symbolic language in the human sense, but they participate in highly organised biological signalling systems. Beyond the empirical characterisation of signalling processes, an additional conceptual issue is whether these processes can properly be described as “communication”. This distinction has been the subject of extensive discussion within the philosophy of biology and biosemiotics, where the relationship between signalling, information, and intentional communication remains controversial [15,17]. If communication is understood as the transmission of information that alters the behaviour of other organisms, then plants do indeed communicate. If, on the other hand, it requires conscious intentionality or symbolic representations, the term becomes problematic.

Plant communication should not be interpreted in anthropomorphic terms, as if plants spoke in the human sense of the word. However, it is clear that they use chemical signals to influence the behaviour of other organisms and coordinate their own responses. The subsoil, too, is a space of extraordinary biological complexity. Mycorrhizal associations between roots and fungi form underground networks through which nutrients and signals are transferred [18]. Through these connections, plants can share carbon, nitrogen and information relating to environmental stress. Some studies even suggest that larger individuals may support younger plants through the transfer of nutrients. Some authors have described these networks as a sort of “wood wide web”, emphasising their cooperative nature [19,20]. Larger trees appear to support younger individuals through the transfer of resources; stress signals can propagate through the shared root system. Although such interpretations are sometimes overly speculative, they highlight an important theoretical point: plants cannot be understood as isolated individuals. The plant organism is intrinsically relational. This conception challenges the individualistic paradigm of classical biology and aligns with ecological and systemic models in which life emerges from networks of interdependence. Biological identity appears to be distributed between the organism and its environment. Thus, the so-called “wood wide web” serves as an effective metaphor for describing ecosystems characterised by profound interdependence. Plant behaviour cannot be understood exclusively at the individual level; rather, it emerges from the continuous interaction between different organisms within complex ecological networks.

## 3. Evolutionary Theory and Plant Adaptation

The evolutionary interpretation of plant behaviour constitutes one of the main theoretical models in contemporary biology, clearly highlighting the complex nature of plant individuals within complex ecological networks, as discussed in the previous paragraph. The theory of natural selection allows us to understand how plants’ adaptive strategies have emerged through gradual processes of functional optimisation, shaped over millions of years by environmental pressures. In the classical formulation of Darwinism, adaptation is interpreted as the result of the action of natural selection on random heritable variations. Organisms possessing advantageous characteristics in a given environment tend to produce a greater number of offspring, leading in the long term to the spread of these traits throughout the population. Plants constitute one of the most extraordinary examples of the explanatory power of this model. The remarkable diversification of angiosperms, the emergence of highly specialised reproductive strategies, and co-evolution with pollinating insects serve as paradigmatic examples of evolutionary adaptation. This phenomenon cannot be viewed as merely a passive reaction to the environment, which is considered an external and immutable reality to which organisms must simply adapt [21,22]. Plants display a unique ability to actively modify the ecological conditions in which they live. Through shading, the production of chemicals, the modification of soil structure and interaction with microbial communities, they contribute to the construction of their own ecological niches [23].

For a long time, evolution was interpreted primarily in relation to animals. Mobility, predatory behaviour, sexual competition and the presence of complex nervous systems seemed to represent the prime arena of evolutionary adaptation. Plants, on the other hand, were regarded as relatively simple organisms, whose evolution appeared to be linked above all to passive morphological changes. However, starting with Darwin’s theory of evolution and continuing with contemporary research, it has been shown not only that this distinction is profoundly reductive, but also that the plant world itself is an example of complex and active evolutionary adaptation, as plants face extremely complex ecological problems and develop sophisticated adaptive strategies to resolve them. Their apparent immobility does not constitute an evolutionary limitation, but rather the starting point for the emergence of alternative modes of adaptation. The condition of sessility—that is, the inability to move through space to escape environmental changes—has exerted a decisive selective pressure in the evolutionary history of plants. Unable to migrate rapidly to favourable environments, they have had to develop strategies based on structural flexibility, physiological plasticity and the ability to modify their own development in response to their environment, as well as to modify the environment itself.

The niche construction theory, developed by Richard Lewontin and subsequently by F. John Odling-Smee, Kevin Laland and Marcus Feldman, has emphasised that organisms are not merely the objects of reactive adaptation, but also agents that transform the selective environment [24,25]. The relationship between organism and environment takes on a circular nature: the environment influences the evolution of organisms, but organisms in turn modify the environment, altering future selective pressures. Once again, plants constitute a particularly significant example of this dynamic. Entire ecosystems—forests, grasslands and wetlands—can be interpreted as the products of the cumulative activity of successive generations of plant organisms. In this sense, the importance of phenotypic plasticity comes to the fore once again, as it represents one of the most significant evolutionary innovations in the plant kingdom. In the case of plants, plasticity is particularly evident in the regulation of growth. A single species can take on profoundly different forms depending on the availability of light, water and nutrients. In shaded environments, for example, many plants develop broader, thinner leaves to increase light absorption; in arid environments, by contrast, they tend to reduce leaf area and invest more in root development. These modifications are not merely mechanical reactions, but adaptive strategies selected over the course of evolution. Plants continuously adjust their energy balance, allocating resources between growth, reproduction and defence in response to environmental conditions. One of the most fascinating aspects of plant evolution concerns precisely this allocation of resources. Every organism has a limited amount of energy, which must be distributed amongst various biological functions. Investing in growth means diverting resources away from defence; investing in reproduction can reduce the ability to withstand environmental stresses. Such evolutionary trade-offs constitute one of the fundamental principles of the theory of evolutionary adaptation. Plants’ behavioural strategies continually reflect this dynamic balance, which underlies evolution. In environments characterised by intense competition for light, many species develop accelerated vertical growth. This phenomenon, known as shade avoidance syndrome [26,27], is regulated by the perception of variations in the light spectrum caused by the presence of other plants. Through phytochromes, plants detect a decrease in red light and activate responses that promote stem elongation. This strategy increases access to light but also entails considerable costs. Excessively rapid growth can, in fact, reduce structural stability and increase vulnerability to water stress. The adaptive response therefore arises from an evolutionary balance between advantages and disadvantages. Defence systems also clearly demonstrate the role of natural selection in the evolution of plant behaviour. Plants have developed an extraordinary variety of chemical and structural strategies to counter herbivores and pathogens. Many species produce alkaloids, tannins, terpenes and other toxic or repellent substances. Others develop thorns, lignified tissues or particularly robust defensive structures. The production of defences entails a high energy cost. Consequently, plants must modulate their defensive investment in relation to the probability of attack. Numerous studies have shown that the synthesis of defensive substances increases significantly following an attack by herbivores [16,28]. This suggests that plants do not keep all defence mechanisms constantly active but dynamically regulate their response according to environmental risk. This aspect reveals a fundamental element of evolutionary adaptation: the ability to optimise the use of resources and thus actively respond to external circumstances.

The evolution of symbiotic relationships also provides a further example of the adaptive complexity of the plant world. Mycorrhizal associations between roots and fungi—already mentioned in the previous paragraph—constitute one of the most important evolutionary events in the history of terrestrial plants. Through these symbiotic relationships, plants increase their ability to absorb nutrients and water, whilst the fungi receive carbohydrates produced through photosynthesis. This cooperation enabled the earliest plants to colonise terrestrial environments that were relatively nutrient-poor. From an evolutionary point of view, mycorrhizal networks demonstrate that natural selection does not exclusively favour competition but can also promote forms of ecological cooperation. Another key theme concerns the adaptation of plants to climate change [29,30]. Throughout their evolutionary history, plant organisms have faced profound environmental changes, developing extremely sophisticated resilience strategies. The emergence of drought-tolerance mechanisms, the regulation of stomatal opening, seed dormancy and the ability to synchronise flowering with seasonal variations are all examples of adaptations selected in response to environmental pressures.

Within the complex dynamics described so far—which also, and above all, characterise plant evolution—lies the concept of co-evolution. Many characteristics of plants can only be understood in the light of evolutionary processes involving several species simultaneously. The relationship between flowering plants and pollinators is one of the best-known examples. The shapes, colours and scents of flowers are often the result of a long history of mutual adaptation between organisms belonging to different evolutionary lineages [31].

Darwin himself had recognised the importance of such phenomena. In his study of orchids, he demonstrated how the structure of certain flowers was inseparable from the morphology of pollinating insects [32]. Evolution therefore does not proceed through isolated organisms, but through networks of ecological relationships that mutually influence one another over time. Once again, recent research on mycorrhizal symbiosis has further reinforced this relational view of evolution. The colonisation of the land by the first plants, some 470 million years ago, is thought to have been made possible by their collaboration with fungi capable of facilitating the absorption of nutrients. The evolutionary identity of plants appears inseparable from the relationships they maintain with other organisms.

Therefore, if evolution is a fundamentally relational process, then biological individuality must also be rethought. Plants demonstrate particularly clearly that an organism is not a self-sufficient entity, but a node within a network of ecological and historical interactions. Adaptation does not concern the survival of isolated individuals solely, but rather the continuous transformation of systems of relationships involving organisms, populations and ecosystems. Whilst not calling into question the central role of natural selection, the importance of the interaction between development, environment and plasticity has been highlighted. Plants therefore constitute a prime model for understanding the dynamic nature of evolution. Indeed, they show that adaptation does not simply consist in the survival of the fittest, but in the emergence of flexible strategies capable of responding to ecological complexity. Evolutionary theory also helps to overcome the traditional dichotomy between active animals and passive plants. Both groups develop strategies aimed at optimising fitness, albeit through profoundly different mechanisms. In the case of plants, behaviour emerges from networked, modular processes that are deeply integrated with the environment. It is precisely this specificity that makes the plant world one of the most fascinating fields in contemporary biology.

The notion of plant behaviour is useful only if it is defined operationally in terms of observable and measurable responses, without presupposing subjective mental states or psychological capacities. Plant behaviours include directional responses, temporal modulations, prioritisation of conflicting signals, changes in the growth space, signal emissions and physiological reorganisation that depend on the context and develop over time. In this sense, behaviour does not equate to visible movement or a mental state; rather, it encompasses processes in which the plant perceives, integrates, selects a response and modifies its ecological trajectory [33].

## 4. Memory, Learning and Adaptation

One of the most innovative and controversial aspects of contemporary research into plant behaviour concerns the possibility that plants exhibit forms of memory and learning. For a long time, such capacities were considered the exclusive preserve of organisms possessing a central nervous system. Memory was interpreted as the result of complex neuronal processes, whilst learning was associated with behavioural modification through experience. Plants, lacking neurons and incapable of voluntary movement, therefore seemed to be excluded from this biological realm. However, numerous recent studies have progressively challenged this view, demonstrating that plant organisms possess sophisticated abilities to record environmental experiences and modulate future responses [34,35,36]. Of course, speaking of plant memory does not mean attributing forms of consciousness analogous to those of humans to plants. The issue is rather to understand whether memory should be defined exclusively in relation to nervous systems, or whether it can be interpreted more broadly as a biological capacity to retain information relating to previous experiences. From this perspective, plants appear to exhibit numerous phenomena attributable to forms of physiological memory.

One of the best-known examples is provided by experiments on *Mimosa pudica*. Normally, this plant reacts to mechanical stimuli by rapidly closing its leaves, a behaviour that reduces the risk of damage from herbivores. Studies conducted by Monica Gagliano and her colleagues have shown that, when repeatedly subjected to harmless stimuli, the Mimosa tends to progressively diminish its defensive response: it reduces the closure of its leaves and maintains this response for prolonged periods. This phenomenon has been interpreted as a form of habituation and physiological memory [34]. The epistemological question is whether such processes can be defined as genuine learning or not. In the most basic sense, learning means permanently altering behaviour on the basis of experience. From this point of view, certain plant phenomena would meet this criterion. If the phenomenon of habituation is traditionally regarded as one of the simplest forms of animal learning, then its presence in plants suggests that even organisms without a brain can modify their behaviour on the basis of experience. Of particular interest is the fact that the modified response persists over time. This implies that the plant is not merely reacting to the present stimulus but retains a physiological trace of the previous experience. This ability to retain information is one of the key factors fuelling the contemporary debate on plant cognition. From a biological perspective, plant memory appears to be based on processes that are profoundly different from neuronal ones. Plants utilise biochemical networks, epigenetic modifications, changes in hormone concentrations and alterations in gene expression to record the effects of environmental experiences.

One of the most widely studied phenomena is so-called defensive priming [37,38]. As mentioned earlier, when a plant is attacked by pathogens or herbivores, certain defence systems are activated and subsequently remain in a state of heightened sensitivity. If the organism is exposed to a similar stressor again, the response will be faster and more effective. This mechanism represents an extremely advantageous adaptive strategy from an evolutionary perspective. Constantly activating all available defences would, in fact, require an enormous expenditure of energy. Through priming, however, the plant can optimise the use of resources whilst maintaining a state of selective readiness. Plant adaptation thus emerges not only as an immediate response to current stimuli, but as the ability to utilise information derived from past experience [16,39]. The ecological memory of plants is also particularly significant in relation to climate change and environmental variability, as I have already seen in relation to evolutionary adaptation. Plants are organisms that are highly exposed to seasonal fluctuations and changes in their ecological context. The ability to record information relating to past events allows them to synchronise their development more effectively. A significant example concerns vernalisation, a process through which certain plants acquire the ability to flower only after being exposed to low temperatures for a specific period. In this case, the organism retains a sort of physiological memory of winter, which enables it to regulate flowering at the most favourable time from a reproductive perspective. Seeds display sophisticated adaptive capabilities linked to environmental memory. Many species are able to remain dormant until specific conditions of temperature, humidity or light indicate the presence of an environment favourable to germination.

All these mechanisms demonstrate how plant behaviour is closely intertwined with temporal processes. Plants do not simply react passively to their current environment but continuously integrate information relating to the past and future probabilities. The role of epigenetics is also particularly significant. Recent studies have shown that certain environmental experiences can produce stable changes in gene expression without directly altering the DNA sequence [40,41]. These epigenetic modifications can, in some cases, be passed on to subsequent generations, influencing the adaptive response of the offspring. This implies that individual experience can indirectly contribute to evolutionary processes. As I have already mentioned in the previous paragraph, although these phenomena do not call into question the fundamental principles of natural selection, they do suggest a more dynamic and complex view of biological adaptation. Plants demonstrate that evolution does not depend solely on random mutations selected over the long term, but also on the ability of organisms to modulate their own development in response to the environment. This perspective has profound theoretical implications. Traditionally, the distinction between physiology and behaviour was relatively clear-cut: behaviour was associated with coordinated and purposeful actions, whilst physiology concerned automatic internal processes. In the case of plants, however, this distinction is gradually becoming blurred. Growth, development, metabolic regulation and ecological adaptation constitute deeply integrated aspects of a single dynamic system. Plant learning, although radically different from that of animals, thus shows that the ability to utilise experience does not necessarily require neurons and synapses. This does not mean that plants think or are conscious in the human sense of the term. They demonstrate that biological memory can take on far more diverse forms than traditionally imagined.

Ultimately, the study of plant memory helps to redefine the very meaning of adaptation. Adaptation no longer appears as a mere passive response to environmental stimuli, but as a dynamic process through which the organism continuously integrates perception, experience and the regulation of development. Plants thus emerge as highly complex biological systems, capable of using the past to modulate their relationship with the future. In this context, the concept of internal self-regulation takes on particular importance. Originally proposed as a biological theory by Maturana and Varela, the concept of autopoiesis has since been developed within the fields of philosophy of biology and systems theory. It provides a useful conceptual framework for interpreting plant self-regulation [42]. Plant behaviour cannot be interpreted merely as a series of episodic responses to environmental stimuli but must be understood as the result of continuous processes of dynamic regulation involving the entire organism. Every plant constantly maintains an extremely delicate internal balance, regulating growth, metabolism, resource allocation and stress response through sophisticated feedback systems. Light, water availability, temperature, nutrients and attacks by pathogens simultaneously influence the organism, which must integrate different and sometimes conflicting signals. Self-regulation represents the plant’s ability to coordinate this information whilst maintaining the functional stability of the biological system. In physiological terms, this process corresponds to a form of dynamic homeostasis, which is fundamental to the survival of plant organisms in variable environments [43]. Plant memory is not merely about the passive retention of traces of experience but constitutes an essential component of the processes of homeostasis and adaptation. Previous experiences do alter the way in which the organism interprets subsequent stimuli, contributing to the regulation of future physiological responses. A significant example is stomatal regulation. Studies by Taiz and Zeiger show that the control of stomatal opening and closing is one of the main mechanisms through which plants regulate the balance between photosynthesis, transpiration and water conservation [7]. Stomata control gas exchange and water loss through transpiration. Under water stress conditions, plants can progressively modify stomatal sensitivity based on previous environmental experiences. This allows them to optimise the balance between carbon dioxide uptake and water conservation, thereby improving their ability to survive. Energy metabolism is also continuously self-regulated. During periods of nutrient or light scarcity, the plant redistributes its resources, slowing down certain growth processes and prioritising functions deemed essential. This capacity for internal reorganisation demonstrates that plant behaviour does not depend on isolated individual reactions, but on the coordinated interaction of multiple physiological systems. Self-regulation is particularly evident in the roots. They do not grow haphazardly in the soil, but constantly adjust their direction, growth rate and branching in response to available environmental information. Roots integrate chemical signals, changes in moisture levels, the presence of physical obstacles and competition with other organisms, dynamically regulating their own architecture. In this sense, the root system can be interpreted as a networked adaptive structure, capable of exploring the environment and continually modifying its strategies. Hormonal regulation also plays a central role in internal self-regulatory processes. Plant hormonal networks represent extremely sophisticated integrative systems, capable of coordinating growth, development and responses to environmental stresses through feedback mechanisms [44]. Auxins, cytokinins, abscisic acid, gibberellins and ethylene form highly integrated signalling networks, through which the plant coordinates growth, defence and development. These networks operate through complex positive and negative feedback mechanisms. A local change can rapidly propagate throughout the entire organism, generating coordinated responses. This aspect once again highlights the distributed nature of plant organisation.

Unlike animals, plants do not possess a central organ responsible for controlling vital functions. Self-regulation arises instead from the continuous interaction between multiple components. From a theoretical point of view, this organisation is reminiscent of contemporary models of complex systems and autopoiesis. According to Capra and Luisi, living organisms must be interpreted as self-organising networks in which stability emerges from the continuous interaction between the components of the system [45]. The plant organism thus appears as a dynamic network capable of maintaining its own identity through continuous processes of self-regulation. Memory becomes an integral part of the organism’s ability to preserve internal stability whilst remaining open to environmental changes. Adaptation therefore does not consist of a simple mechanical reaction to external stimuli, but of a continuous negotiation between the environment, past experience and internal regulation. This perspective contributes to a profound redefinition of the very concept of plant behaviour. Plants no longer appear as rigidly programmed organisms, but as dynamic biological systems capable of integrating memory, perception and self-regulation into highly sophisticated adaptive strategies.

Within this framework, evolutionary theory is not an external addition, but the criterion that distinguishes the most convincing cases from the more speculative ones. If a behaviour is repeatable, mechanistically grounded, linked to differences in fitness and comparable across species or populations, then it can be treated as a trait subject to selection. When the terminology exceeds the empirical evidence—as in the attribution of consciousness or fully animal-like associative learning—the recent literature urges caution. This review does not argue for plant consciousness. Rather, it addresses whether certain plant behaviours satisfy the operational criteria for cognition, regardless of subjective experience. As persistence of a physiological modification is not sufficient to establish learning or cognitive memory, the above phenomena should be distinguished according to their empirical status and conceptual implications. Table 1 summarises this evidential hierarchy and indicates the appropriate terminology for each phenomenon. The table distinguishes the empirical status of selected plant phenomena from the terminology appropriate to their description and from the strength of the cognitive interpretation that they may support. Persistent physiological or developmental modification is not treated as sufficient evidence of cognition or learning. Stronger cognitive interpretations require additional evidence of context-sensitive integration, flexible response modulation, and the use of information acquired through previous interactions.

This hierarchy is important because persistence alone is not enough for cognition. A physiological state can retain the effects of previous environmental conditions without constituting learning. Therefore, vernalisation, dormancy, priming and epigenetic inheritance should not be indiscriminately grouped under cognitive memory. A stronger cognitive claim requires evidence that retained information modifies subsequent context-sensitive behaviour in a way that satisfies independently specified learning criteria.

## 5. The Debate on Plant Intelligence

Throughout this section, the terms “cognition” and “intelligence” are used in distinct senses. “Cognition” refers to the processes by which an organism integrates biologically relevant information and modulates its subsequent responses according to its state, previous interactions and environmental conditions. In contrast, intelligence is used more narrowly to denote flexible, adaptive problem solving when more than one possible response is available. According to this definition, intelligence may be considered a manifestation of cognition, but the two terms are not synonymous. Furthermore, neither cognition nor functional intelligence, as used here, implies phenomenal consciousness. Everything that has emerged and that I have discussed in the preceding paragraphs—namely, the fact that plants are undoubtedly highly plastic, sensitive and informationally integrated biological systems, and that they exhibit their own specific behaviour—has opened up the topic of plant intelligence. It must be made clear from the outset that the debate on plant intelligence does not centre solely on a biological question—whether plants learn, remember, make decisions or act—but on a question of the philosophy of biology: which concepts of cognition, mind, agency and behaviour can legitimately be extended beyond animals with a nervous system. In recent decades, the growing focus on plant behaviour has led many scholars to question whether organisms lacking a nervous system might exhibit forms of cognition, learning and decision-making [13,35,36]. The main difficulty stems from the fact that the concept of intelligence is historically and culturally associated with animals, and in particular with human beings. The mechanisms involved in experience-dependent modifications of plant responses are heterogeneous and may include hormonal and calcium signalling, changes in gene expression, and transcriptional and metabolic regulation. In some cases, they may also include epigenetic modifications. Therefore, the conceptual question addressed here concerns not any single molecular mechanism, but rather whether the coordinated operation of such physiological and signalling processes can justify the use of cognitive terminology under specified conditions. The biological complexity of these processes is not enough on its own; the important issue is whether they support context-sensitive integration and the flexible modulation of subsequent responses based on information acquired through previous interactions with the environment. Traditionally, intelligence has been defined in relation to the presence of a brain, specialised neurons and centralised cognitive processes. Applying this category to plants implies a profound re-evaluation of traditional interpretative frameworks, given that for much of the history of modern science, the plant world has been described through a mechanistic paradigm. The historical lineage of those who advocate the idea that I can speak of plant intelligence at various levels can be traced back to Darwin’s statement, according to which the tip of a plant’s radicle acts like the brain in animals [5]. It must be said that this analogy does not, in itself, amount to modern evidence of plant cognition; nevertheless, it has shaped the imagination and vocabulary of contemporary debate. Proponents of a strong thesis interpret this Darwinian statement as proof that Darwin himself believed plants possessed plant intelligence. Hence the idea that plants are intelligent in a literal sense because they integrate signals, display goal-directed plasticity, memory, learning and distributed forms of information without a centralised brain. Among the most influential figures in this field are Anthony Trewavas, František Baluška, Stefano Mancuso and, more radically, Monica Gagliano. They have contributed significantly to research on plant learning and consciousness.

In current terminology, plant intelligence and plant cognition are not synonymous. Intelligence is often used in a broad functional sense—the capacity to modify behaviour to improve survival and fitness under real-world conditions. Cognition is a more technical and theoretically charged term. For some, it requires representations or computation; for others, it can be understood in enactive, ecological or biogenic terms, or as a fine-grained concept comprising partial capacities. It is precisely this plurality of definitions that is one of the reasons why the debate persists [35]. The interpretation of these phenomena largely depends on the theoretical conception of cognition adopted. According to Anthony Trewavas, intelligence should be defined not on the basis of the presence of specific anatomical structures, but in functional terms [3,36]. An organism can be considered intelligent to the extent that it is capable of perceiving environmental information, integrating it, modifying its behaviour and solving adaptive problems. As I have seen in the previous paragraphs, plants appear to meet many of these criteria. They simultaneously perceive a wide range of stimuli, integrate information from different sources and modulate their own development in response to the ecological context. Root growth is a particularly significant example. Roots do not develop randomly in the soil, but explore the environment selectively, orienting themselves towards areas richer in water and nutrients and avoiding unfavourable conditions. Furthermore, Trewas’s thesis is explicitly anti-neurocentric. Intelligence would not, in principle, require neurons or a brain. The position taken by Mancuso and Baluska is stronger—and therefore also more controversial—as they use more explicit language. Mancuso defended the programme of plant neurobiology as an integrated study of signalling, communication and plant behaviour, drawing on long-distance electrical signals, analogies with networks and even metaphors such as “plant synapses” or “root-brain” [10]. The heuristic power of this vocabulary has been considerable, although there is a danger of over-relying on analogies. Since plants do not possess a nervous centre, but the entire organism participates in information processing, according to Stefano Mancuso, such behaviour demonstrates the existence of forms of decentralised problem-solving [52]. This distributed organisation represents one of the most innovative elements of the contemporary debate. In higher animals, behaviour is generally coordinated by a central brain. Plants, on the other hand, function through networks in which every part of the organism contributes to overall regulation. This is what is meant by distributed intelligence. Cognition would not necessarily depend on the presence of neurons but could emerge from complex systems capable of integrating information and self-regulating. The theory of complex systems has played an important role in the development of these interpretations. According to Capra and Luisi, many biological systems exhibit emergent properties that cannot be explained solely through the analysis of their individual components [45]. Plants would therefore represent a form of cognitive organisation radically different from that of animals, but no less sophisticated for that.

The disputes seem to concern not so much the empirical evidence itself, but rather the interpretative and semantic framework through which I refer to that evidence. One example is the phenomenon of systemic signalling. In *Arabidopsis thaliana*, Toyota and colleagues have shown that glutamate acts as a wound signal and that GLR channels convert this signal into calcium waves that propagate to distant organs, inducing defensive responses. The recent literature confirms the importance of rapid electrical signals for the coordination of the entire plant. There is no substantial controversy regarding the phenomenon itself: the controversy rather concerns whether to describe it as information processing in a cognitive sense or as sophisticated but non-mental physiology [28]. There are many other important examples that support the idea that plants discriminate between complex environmental signals. *Arabidopsis* modifies its defences when exposed to vibrations produced by herbivore chewing, distinguishing them from other vibrations. *Cuscuta pentagona* uses volatile compounds to locate hosts and assess their attractiveness. Even in this case, however, the transition from selective sensitivity to cognition is not automatic. Much depends on how broad or restrictive the theory of cognition adopted is [53]. In this regard, an emblematic position is that of Monica Gagliano, with her studies on learning in *Mimosa pudica*, to which I have already referred in the context of the physiological memory of plants [54]. The researcher argued that repeated harmless falls induced habituation of the plant’s leaf-closing response, with a persistence that she interpreted as an elementary form of learning and memory. Thus, accepting this line of interpretation, the experiments on habituation would have shown that plants can permanently modify their behaviour on the basis of experience. These results suggest that learning is not the exclusive preserve of nervous systems. Far more controversial is the case of associative learning in peas. In 2016, Gagliano and colleagues reported, in a Y-paradigm, that *Pisum sativum* could associate airflow with a future light position, directing its growth towards the neutral “cue” [50]. In 2020, Markel attempted a replication using larger, blinded samples and found no evidence of the effect. This was followed by comments and further replications, with disagreement over differences in protocol [51]. Philosophically, this episode shows just how much the field depends on methodological details, pre-registration, observer blindness and standards of replicability comparable to those in comparative psychology.

The debate on plant intelligence has taken on a strong philosophical and epistemological dimension. However, one of the main problems concerns the risk of anthropomorphism—that is, interpreting processes for which less mentalistic explanations exist as choices, intentions, altruism or motherhood. The recent critique of the personification of mycorrhizal networks and mother trees is instructive in this regard [55]. Numerous scholars argue that using terms such as intelligence, decision-making, or memory can be misleading, as these categories derive historically from human and animal experiences. For example, Alpi and other colleagues challenged the legitimacy of plant neurobiology, arguing that, in the absence of a brain and nerves, neurobiological terminology causes more confusion than theoretical progress. Plants do not possess neurons, synapses or brains. Consequently, attributing cognitive abilities to them risks confusing physiological phenomena with mental processes [56]. In turn, Taiz put forward an even more specific critique, arguing that the adaptive complexity of plants is not evidence of consciousness. Plant evolution has followed an alternative fitness pathway, based on genetically and epigenetically regulated plasticity rather than on subjective experience.

At the same time, I must also avoid falling into the opposite trap—that of reducing any observed flexibility to mere hard-wired routines, whilst ignoring the multi-scale integration of plant behaviour. The most promising positions today are those that do not seek to establish once and for all whether plants are cognitive but rather invite us to break the problem down into more circumscribed capacities: information integration, decision-making, memory, anticipation, selective sensitivity and sensorimotor coordination. This line of thought is represented above all by Paco Calvo, Miguel Segundo-Ortin, Jonny Lee, David Colaço and, in Italy, Margherita Bianchi [13,57]. These authors tend to view plant cognition as a borderline case useful for reformulating the concept of cognition in a biogenic, enactive, ecological or piecemeal sense. The debate thus functions as an exploratory conceptual hypothesis. The most appropriate course of action is always to operationalise before interpreting. If one is studying learning, one must specify what type of learning: habituation, sensitisation, classical conditioning, priming? If one is studying memory, one must establish the relevant temporal and mechanistic basis. If decision-making is invoked, one must define the alternatives, the available information, the metric of choice, and the control for simpler explanations. This is precisely the thrust of the recent methodological approach that links cognitive theory, the philosophy of science and comparative psychology in the study of plants. Furthermore, it is important to link the behavioural level to the mechanical level without reducing the former to the latter, by combining, for example, the phenomenology of behaviour, calcium imaging, electrophysiology, hormones, high-resolution phenotyping and modelling. In the lexicon of the philosophy of biology, I need bridging explanations between emergent traits and underlying causal networks, not slogans or mere inventories of correlations.

Rather than asking whether plants are intelligent in an absolute sense, it is more productive to evaluate which concepts are justified by the available empirical evidence. While some plant phenomena are uncontroversially behavioural, others satisfy operational criteria for memory or adaptive information integration. Much stronger claims concerning cognition or intelligence, however, remain dependent upon the theoretical framework adopted. Therefore, the debate concerns not only biological evidence, but also the explanatory scope of the concepts used to interpret it. Therefore, the debate on plant intelligence should not be framed as a binary opposition between “plants are intelligent” and “plants are not intelligent”. Instead, it concerns the legitimacy of different conceptual vocabularies applied to increasingly sophisticated empirical evidence. Operational definitions, explicit theoretical commitments, and methodological transparency are therefore essential if future research is to progress beyond terminological disputes. In the present account, terms such as “root brain” and “plant synapse” have, at most, a heuristic value. While they may draw attention to functional similarities, such as distributed signal integration, long-distance coordination, and local information processing, they should not be interpreted as claims of anatomical or mechanistic homology with animal nervous systems. Furthermore, analogy alone has limited explanatory value. Where neuromolecular terminology does not yield experimentally distinguishable predictions beyond those generated by the established concepts of electrical signalling, hormonal regulation, and vascular communication, the latter terminology is scientifically preferable.

## 6. Conclusions

The argument developed in this review therefore favours a graded rather than a binary interpretation of plant capacities. Firstly, attributing behaviour to plants is well supported when behaviour is defined in terms of context-dependent modifications to growth, physiology, or interaction with the environment. Secondly, the term “intelligence” can be used in a limited functional sense to describe flexible, adaptive problem solving, but this usage should not be confused with psychological intelligence. Thirdly, cognition is a stronger and more controversial attribution. According to the non-representational account defended here, only cases involving integrated, context-sensitive, flexible regulation, where previous states or acquired information modify subsequent organism-level responses, are candidates for minimal cognition. Merely being adaptive does not satisfy these criteria for ordinary metabolism, homeostasis, developmental plasticity and physiological self-maintenance. Furthermore, none of the material reviewed here provides sufficient evidence to attribute phenomenal consciousness or subjective experience to plants. Therefore, the resulting position is neither that plants are intelligent in an animal-like sense, nor that all plant adaptation should be reclassified as cognition. A more defensible conclusion is that a subset of plant phenomena constitutes a legitimate test case for minimal, non-conscious, non-representational cognition. Whether individual phenomena cross that threshold must be established experimentally rather than inferred from biological complexity alone. Future progress will consequently require experiments designed not merely to demonstrate complex plant responses, but also to discriminate between competing explanations of these responses. Priorities include:

- Preregistered and adequately powered replications of controversial learning experiments;
- Standardised experimental protocols;
- Blinded behavioural assessment;
- Explicit reporting of effect sizes and uncertainty;
- Comparative studies across species and populations;
- Experiments that directly contrast cognitive hypotheses with simpler physiological alternatives.

This approach would shift the focus of the debate from disputes over terminology to experimentally tractable questions concerning the organisation and evolution of adaptive control in living systems. The controversy surrounding plant intelligence ultimately does not arise from conflicting empirical observations, but from the different conceptual frameworks used to interpret them. The same experimental evidence may lead to different conclusions, depending on whether intelligence is defined as neural computation, adaptive problem solving, information integration or embodied cognition. Rather than asking whether plants are “really intelligent”, it is more productive to ask which definition of intelligence best captures the biological phenomena under investigation. From this perspective, the debate shifts from asking a simple yes-or-no question to conducting a conceptual analysis of the explanatory value of different theoretical frameworks. However, intelligence may still be used in a limited, functional sense to describe flexible, adaptive problem solving, provided this usage is clearly distinguished from stronger, more mentalistic conceptions involving conscious or representational capacities.

## Figures and Tables

**Table 1:** Evidential and conceptual status of selected forms of information retention and experience-dependent modification in plants.

Phenomenon	Empirical Status	Appropriate Terminology	Cognitive Implication	References
Vernalisation	Well established	Developmental/physiological memory	No cognitive implication by itself	[7]
Seed dormancy	Well established	Developmental regulation/environmental information retention	No cognitive implication by itself	[46]
Defensive priming	Well established	Physiological memory/priming	Compatible with minimal memory, but insufficient by itself to establish cognition	[16,39,47]
Stress-induced epigenetic modification	Well established in many experimental contexts	Epigenetic regulation/stress memory	No cognitive implication by itself	[40,48]
Transgenerational epigenetic inheritance	Established in specific cases, but scope and stability vary	Transgenerational epigenetic inheritance	Does not constitute learning by itself	[40,49]
Habituation-like response in *Mimosa pudica*	Empirically reported, but interpretation remains debated	Candidate habituation/experience-dependent response modification	Potential evidence for minimal learning if alternative physiological explanations are excluded	[34]
Associative learning in *Pisum sativum*	Disputed; original finding has not been robustly replicated	Candidate associative learning	Insufficient evidence at present for a cognitive conclusion	[50,51]
Systemic wound signalling	Well established	Long-distance electrical and Ca^2+^-based signalling	Evidence of integrated physiological information processing, but not cognition by itself	[28]
Context-sensitive root growth/foraging	Well established as developmental plasticity	Adaptive growth regulation/context-sensitive plasticity	Possible candidate for minimal cognition only under additional criteria of flexible response selection	[3,13]

## Data Availability

No new data were created or analyzed in this study. Data sharing is not applicable to this article.
